# WU Polyomavirus in Respiratory Epithelial Cells from Lung Transplant Patient with Job Syndrome

**DOI:** 10.3201/eid2101.140855

**Published:** 2015-01

**Authors:** Erica A. Siebrasse, Diana V. Pastrana, Nang L. Nguyen, Annie Wang, Mark J. Roth, Steven M. Holland, Alexandra F. Freeman, John McDyer, Christopher B. Buck, David Wang

**Affiliations:** Washington University School of Medicine, St. Louis, Missouri, USA (E.A. Siebrasse, N.L. Nguyen, A. Wang, D. Wang);; National Institutes of Health, Bethesda, Maryland, USA (D.V. Pastrana, M.J. Roth, S.M. Holland, A.F. Freeman, C.B. Buck);; University of Pittsburgh Medical Center, Pittsburgh, Pennsylvania, USA (J. McDyer)

**Keywords:** WU polyomavirus, polyomavirus, viruses, lung transplant, immunosuppression, respiratory epithelial cells, tropism, lung transplant recipient, Job syndrome

## Abstract

We detected WU polyomavirus (WUPyV) in a bronchoalveolar lavage sample from lungs transplanted into a recipient with Job syndrome by using immunoassays specific for the WUPyV viral protein 1. Co-staining for an epithelial cell marker identified most WUPyV viral protein 1–positive cells as respiratory epithelial cells.

WU polyomavirus (WUPyV) was discovered in a child with pneumonia in 2007 (*1*). Subsequent studies showed that WUPyV infection is common (*2**–**4*), and viral DNA can be detected in a variety of specimen types, including respiratory tract secretions (*5*). However, the virus has yet to be associated with any disease, and the specific cell type(s) infected by WUPyV has not been identified. Other human polyomaviruses are known pathogens, which typically cause disease in the context of immunosuppression (*6**–**8*).

Job syndrome is an immune disorder characterized by eczematoid dermatitis, recurrent skin and pulmonary infections, increased levels of IgE, and impaired T and B cell memory (*9*). This disorder is caused by dominant-negative mutations in the *STAT3* gene (*9*). We report WUPyV cell tropism in lungs transplanted into a recipient with Job syndrome.

## The Study

These studies were approved by institutional review boards at the National Institutes of Health (NIH) and Washington University. A 28-year-old woman with Job syndrome was seen at the NIH Clinical Center 6 months after bilateral lung transplantation. She had a bronchoscopic evaluation to follow up on endobronchial aspergillosis. Pathologic examination of a bronchoalveolar lavage (BAL) sample showed scattered cells, primarily columnar bronchial cells, with cytomorphologic changes reminiscent of BK polyomavirus (BKPyV)–infected decoy cells. The cells stained positive with PAb416, a monoclonal antibody against the SV40 large T antigen. The patient had BKPyV viremia (8.1 × 10^5^ copies/mL) and viruria (6.9 × 10^9^ copies/mL). JC polyomavirus was also detected in the urine but not in the blood. The BAL sample was weakly positive for BKPyV by PCR (<250 copies/mL) and negative for JC polyomavirus. Clinical or radiographic signs and symptoms of infection were not apparent.

Nonenveloped virions were purified from the BAL sample by using ultracentrifugation with Optiprep (#D1556; Sigma-Aldrich, St. Louis, MO, USA) (*10*). DNA was extracted from the virion preparation and subjected to random-primed rolling circle amplification (RCA) and restriction enzyme digestion, which yielded 2 strong bands. The bands were cloned and identified as WUPyV by using Sanger sequencing. The complete genomic sequence of the isolate, designated J1 (GenBank accession no. KJ643309), was confirmed by using miSeq analysis (Illumnina, San Diego, CA, USA) of the RCA product. A second WUPyV variant with 2-nt polymorphisms and a single base insertion was also detected in the RCA product. No other known viruses (including BKPyV) were observed by deep sequencing.

We developed an immunohistochemical (IHC) assay to detect the WUPyV viral protein 1 (WU-VP1) by using an IgG2b designated NN-Ab06. Recombinant histidine-tagged WU-VP1 protein was generated by expressing WU-VP1 (GenBank accession no. ABQ09289) in *Escherichia coli* from a Gateway pDEST17 plasmid (Life Technologies, Carlsbad, CA, USA) and purifying the protein by using an affinity Ni-NTA column (Pierce Biotechnology, Rockford, IL, USA). After generation of hybridomas, we identified clones producing antibody against WU-VP1 by ELISA and immunoblot. Clones that cross-reacted with KI polyomavirus VP1 (KI-VP1) were identified by ELISA with glutathione S-transferase–tagged KI-VP1 (*2*) and eliminated.

To generate positive control cells for IHC assay optimization, we transfected 293T cells with plasmid pDEST26-WU-VP1 (Life Technologies). A subset of cells was fixed in 10% neutral-buffered formalin and embedded in paraffin. IHC testing was performed by deparaffinizing slides in xylene and rehydrating them in a series of ethanol solutions. After treating slides with 3% hydrogen peroxide, antigen was retrieved in citrate buffer, pH 6.0 (10 mmol/L citric acid, 0.05% Tween 20) in a pressure cooker (PC6–25; Nesco, Two Rivers, WI, USA) for 3 min on the high setting.

Slides were blocked in 1.5% normal horse serum (#S-200; Vector Laboratories, Burlingame, CA, USA) and incubated with NN-Ab06, then with biotinylated anti-mouse IgG (BA-2000; Vector Laboratories). After development by using the Vectastain Avidin–Biotin Complex Kit (#PK-6100; Vector Laboratories) and (3,3′-diaminobenzidine) (#SK-4100; Vector Laboratories), we counterstained tissues with hematoxylin.

**Figure 1:**
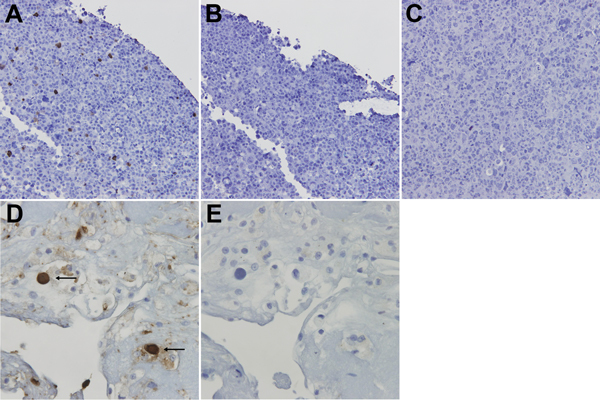
WU polyomavirus antigen in bronchoalveolar lavage specimens from lungs transplanted into a recipient (28-year-old woman) with Job syndrome. Immunohistochemical analysis of 293T cells transfected with pDEST26-WU–virus protein 1 and stained as follows. A) WU virus protein 1 monoclonal antibody (NN-Ab06). B) Isotype control. C) Mock transfected 293T cells stained with NN-Ab06. D) Bronchoalveolar lavage specimen stained with NN-Ab06 showing prominent dark staining of cells with enlarged nuclei and a ground glass appearance characteristic of viral cytopathic changes (arrows). E) Isotype control. Original magnifications ×400 in panels A–C and ×600 in panels D and E.

Cells with prominent dark staining were seen (Figure 1, panel A). A serial section of the same cell block stained with an isotype-matched antibody (#557351, mouse IgG2b: BD Biosciences, San Jose, CA, USA) (Figure 1, panel B) and mock transfected cells stained with NN-Ab06 (Figure 1, panel C) showed negative results. Western blotting was performed as an independent means of evaluating specificity of NN-Ab06 (*11*). NN-Ab06 reacted with WU-VP1 protein lysate but not with KI-VP1 lysate, which is the most closely related virus to WUPyV. KI-VP1 has 65% amino acid identity with WU-VP1.

We applied the WU-VP1 IHC assay to formalin-fixed, paraffin-embedded sections of the BAL sample. Prominent dark staining of cells with enlarged nuclei and a ground glass appearance characteristic of viral cytopathic changes were observed (Figure 1, panel D). Staining was not seen in serial sections stained with the isotype antibody (Figure 1, panel E) or with no antibodies.

**Figure 2:**
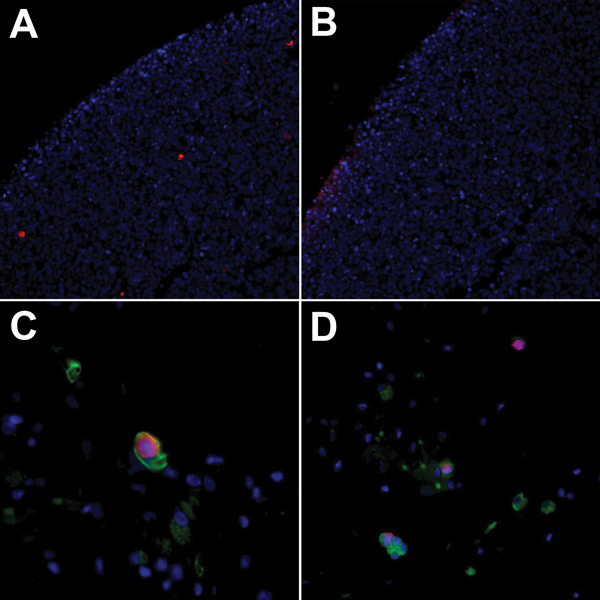
WU polyomavirus antigen in respiratory epithelial cells from lungs transplanted into a recipient (28-year-old woman) with Job syndrome. Immunofluorescence of 293T cells transfected with pDEST26-WU–virus protein 1 and stained with A) WU virus protein 1 polyclonal antibody (NN-Ab01) or B) preimmune serum. C) Double immunofluorescence with NN-Ab01 (red) and a monoclonal antibody against cytokeratin (green) showing a double-positive cell from the bronchoalveolar lavage specimen. D) Bronchoalveolar lavage specimen with multiple WU virus protein 1/cytokeratin double-positive cells. Original magnifications ×100 in panels A and B, ×600 in panel C, and ×400 in panel D.

Many WUPyV-positive cells were cuboidal to columnar and showed other morphologic features consistent with respiratory epithelial cells. To determine their etiology, we developed a double immunofluorescence (dIF) assay with a polyclonal antibody against WU-VP1 (*2*), designated NN-Ab01, and a monoclonal antibody against cytokeratins (#M3515; Dako, Carpinteria, CA, USA). Deparaffinization and antigen retrieval were accomplished as noted above, and sections were blocked in Superblock T20 (#37516; Thermo Scientific, Waltham, MA, USA). To validate the assay, we performed immunofluorescence analysis with NN-Ab01 on positive control 293T cells expressing WU-VP1. The nucleus was counterstained with Hoechst (#H21491; Life Technologies). Several WU-VP1-positive cells were observed (Figure 2, panel A); a serial section stained with preimmune serum at the same dilution showed a negative result (Figure 2, panel B).

For dIF, BAL sections were incubated first with the primary antibodies and then with fluorescently labeled secondary antibodies (#A10042 anti-rabbit-568 and #A10042 anti-mouse-488; Life Technologies). We observed cells positive for WU-VP1 and cytokeratin (Figure 2, panels C, D), which identified these cells as epithelial cells. Of the 136 WU-VP1-positive cells, 77 (57%) were also cytokeratin positive. A serial section of the BAL sample stained with an isotype-matched antibody to the cytokeratin antibody (#555746 mouse IgG1; BD Biosciences) and preimmune rabbit serum was negative.

We hypothesized that the remaining 43% of WU-VP1-positive, cytokeratin-negative cells might be macrophages. However, a dIF assay using NN-Ab01 and an antibody against CD68 (#M0814; Dako), a macrophage marker, showed WU-VP1 and CD68 single-positive cells but no double-positive cells. In addition, a stain with NN-Ab01 and an antibody against CD45 (#M351529–2; Dako), a marker for hematopoietic cells, also showed negative results.

## Conclusions

Before this study, to our knowledge, no specific cell type had been identified as susceptible to WUPyV infection. We found that WUPyV antigen was detected in human respiratory epithelial cells. The presence of nuclease-resistant viral DNA from the Optiprep gradient and detection of WU-VP1, which is believed to be expressed concomitantly with DNA replication (*12*), suggests that the cells were infected by WUPyV and that the WUPyV life cycle reached at least the stage of late gene expression. The clinical role of infection by WUPyV is uncertain, given that the patient was not experiencing any recognizable symptoms. Although we attempted to identify a second population of WU-VP1-positive, cytokeratin-negative cells, the etiologic features of these cells remains uncertain.

Our patient had Job syndrome, a primary immunodeficiency not previously associated with polyomavirus susceptibility. It is possible that immunosuppressant medications, which include prednisone and tacrolimus, altered susceptibility to virus infection. Other human polyomaviruses are believed to exclusively cause disease in immunocompromised hosts. This case suggests that immunosuppression might also play a role in WUPyV infection and expands our understanding of WUPyV biology. 
